# Associations of Lancet Commission risk factors for Alzheimer's disease with blood‐based biomarkers and mild cognitive impairment from midlife to early old age

**DOI:** 10.1002/alz70860_105091

**Published:** 2025-12-23

**Authors:** Carol E. Franz, Erik Buchholz, Tyler R. Bell, Jeremy A. Elman, Christine Fennema‐Notestine, Daniel E. Gustavson, Jack F.V. Hunt, Paula Iso‐Markku, Amy J. Jak, Michael J. Lyons, Nathan A. Gillespie, Michael C. Neale, Ryan O'Leary, Matthew S. Panizzon, Alexander Ivan B. Posis, Chandra A. Reynolds, Robert A. Rissman, Rongxiang Tang, Tina T. Vo, McKenna E. Williams, Arthur Wingfield, William S. Kremen, the Vietnam Era Twin Study of Aging (VETSA)

**Affiliations:** ^1^ University of California San Diego, La Jolla, CA, USA; ^2^ Center for Behavior Genetics of Aging, University of California, San Diego, La Jolla, CA, USA; ^3^ University of Arkansas at Little Rock, Little Rock, AR, USA; ^4^ University of California, San Diego, La Jolla, CA, USA; ^5^ University of Colorado Boulder, Boulder, CO, USA; ^6^ Helsinki University Hospital, Helsinki, Finland; ^7^ Boston University, Boston, MA, USA; ^8^ Virginia Commonwealth Univeristy, Richmond, VA, USA; ^9^ Brandeis University ryoleary@brandeis.edu, Waltham, MA, USA; ^10^ Herbert Wertheim School of Public Health and Human Longevity Science, University of California, San Diego, La Jolla, CA, USA; ^11^ University of California, Davis, Davis, CA, USA; ^12^ University of Southern California, San Diego, CA, USA; ^13^ Texas A&M University, College Station, TX, USA; ^14^ Brandeis University, Waltham, MA, USA

## Abstract

**Background:**

The 2024 Lancet Commission report identified 14 modifiable risk factors associated with dementia prevention that, if addressed, could reduce dementia incidence by as much as 45%. Little attention has been paid to simultaneous examination of all 14 risk factors in relation to Alzheimer's disease (AD) blood‐based biomarkers or mild cognitive impairment (MCI).

**Method:**

We operationalized the 14 risk factors in 936 men in the Vietnam Era Twin Study of Aging at average age 62 when all risk measures were available: education, hearing, vision, depressive symptoms, social engagement, physical exercise, traumatic brain injury (TBI), glucose, low density lipoprotein (LDL), body mass index (BMI), blood pressure, smoking, alcohol consumption, and air pollution (particulate matter 2.5: PM2.5). Outcomes at average age 68 comprised plasma biomarkers [Aβ42/40, pTau231, neurofilament light chain (NfL), glial fibrillary acidic protein (GFAP)] and MCI diagnosis. To address co‐linearity and non‐linearity among predictors, analyses were conducted with Random Forest using grid search and nested cross‐validation, with and without adjustment for age and *APOE* status (ε4+; ε4‐). Outcomes were examined separately.

**Result:**

Risk factors accounted for 0% (ptau231, AB42/40), 7% (NfL) and 8% (GFAP) of the biomarker variance. The AUC for predicting MCI by age 68 was 0.62. The 3 strongest predictors for NfL were fasting glucose, education, and BMI; for GFAP: BMI, education, and PM2.5. For MCI, predictors in descending order of importance were: blood pressure, PM2.5, education, LDL, BMI, glucose, depressive symptoms, TBI, alcohol consumption, exercise, social engagement, hearing, acuity, age, and smoking. When added to the models, *APOE* was the least important contributor to plasma NfL, GFAP, and MCI.

**Conclusion:**

Correlated Lancet Commission risk factors when considered together, accounted for relatively little of the variance in plasma NfL, GFAP, or MCI in men across 6 years from late midlife to early old age. The modifiable risk factors contributed more strongly than *APOE* and/or age. Top predictors for biomarkers and MCI were similar (glucose, BMI, PM2.5, education) illustrating the importance of modifiable health and environmental factors. These results highlight the complex health and lifestyle etiologies of AD‐related plasma biomarkers and may signal areas of opportunity for early intervention.

